# Time Series Multiple Channel Convolutional Neural Network with Attention-Based Long Short-Term Memory for Predicting Bearing Remaining Useful Life

**DOI:** 10.3390/s20010166

**Published:** 2019-12-26

**Authors:** Jehn-Ruey Jiang, Juei-En Lee, Yi-Ming Zeng

**Affiliations:** Department of Computer Science and Information Engineering, National Central University, Taoyuan City 32001, Taiwan; 105522050@cc.ncu.edu.tw (J.-E.L.); 105522116@cc.ncu.edu.tw (Y.-M.Z.)

**Keywords:** bearing, convolutional neural network, deep learning, long short-term memory, remaining useful life, time series

## Abstract

This paper proposes two deep learning methods for remaining useful life (RUL) prediction of bearings. The methods have the advantageous end-to-end property that they take raw data as input and generate the predicted RUL directly. They are TSMC-CNN, which stands for the time series multiple channel convolutional neural network, and TSMC-CNN-ALSTM, which stands for the TSMC-CNN integrated with the attention-based long short-term memory (ALSTM) network. The proposed methods divide a time series into multiple channels and take advantage of the convolutional neural network (CNN), the long short-term memory (LSTM) network, and the attention-based mechanism for boosting performance. The CNN performs well for extracting features from data with multiple channels; dividing a time series into multiple channels helps the CNN extract relationship among far-apart data points. The LSTM network is excellent for processing temporal data; the attention-based mechanism allows the LSTM network to focus on different features at different time steps for better prediction accuracy. PRONOSTIA bearing operation datasets are applied to the proposed methods for the purpose of performance evaluation and comparison. The comparison results show that the proposed methods outperform the others in terms of the mean absolute error (MAE) and the root mean squared error (RMSE) of RUL prediction.

## 1. Introduction

With the advanced development of technologies of sensors, robots, Internet-of-Things (IoT), artificial intelligence (AI), and industrial automation, now comes the era of Industry 4.0 [1]. The vision of Industry 4.0 is to build smart factories that automate and manufacture intelligently to improve product quality, manufacturing efficiency, and production flexibility. The Prognostics and Health Management (PHM) of machines plays a critical role in smart factories, as it can assess the reliability of machines in their life cycles and determine the advent of failures to mitigate risks of sudden machine breakdowns [2]. The study of PHM first started in the medical sciences, and then was introduced into mechanical sciences [3]. Like medical prognostics aiming to predict potential diseases and perform pretreatment for patient health, the PHM of machinery focuses on degradation prediction and maintenance of machines [4].

The machinery remaining useful life (RUL) prediction (or estimation) is one of the most important aspects of PHM. The RUL of a machine is defined as the length from the current time to the time when the extent of deviation or degradation of the machine from its expected normal operating conditions exceeds a threshold [5]. Accurate machinery RUL prediction is useful for replacing/repairing machines before they become faulty. This may prevent great losses caused by sudden stop of machines. There are many methods proposed for accurately predicting the RUL of machines or their components, such as wind turbine gearboxes [6], wind turbine blade [7], train wheels [8], aviation piston pumps [9], aircraft propulsion engines [10], rotary machines [11], and bearings [12], just to name a few.

This paper focuses on the RUL prediction of bearings. Existing bearing RUL prediction methods can be roughly classified as model-based methods and data-driven methods. Model-based methods derive mathematical equations as models that follow physical laws to represent the degradation processes for predicting the bearing RUL. For example, the Paris—Erdogan law [13] for the growth and propagation of fatigue cracks is transformed into a state—space model for predicting the RUL of rotary machines [11]. Based on the model, an improved exponential model along with particle filters was proposed to estimate the RUL of bearings [12]. Model-based methods require expert domain knowledge and they may be too costly and too complex to develop.

Data-driven methods extract features from large volumes of data gathered from sensors and try to find specific relationships between feature patterns and the bearing RUL. For example, principal components analysis (PCA) is first applied to vibration signals of sensors attached to bearings for extracting critical features [14]. Least squares-support vector regression (LS-SVR) is then applied to new PCA features for predicting the RUL of bearings [14]. Unlike model-based methods, data-driven methods depend on little domain knowledge and can be improved significantly in the aspect of prediction accuracy by applying advanced data analysis techniques, such as machine learning schemes and deep learning schemes. Data-driven methods are thus easier to realize than model-based methods, and they have recently been adopted by many researchers. It is worthwhile mentioning that besides data-driven methods for solving the bearing RUL prediction problem, there are many data-driven methods [15,16,17,18,19,20,21,22,23,24,25,26,27,28,29] for solving bearing PHM-related problems, such as bearing fault detection (or identification), fault diagnosis, fault classification, degradation state recognition, etc. The mechanisms used by those methods include the PCA and its variants, linear discriminant analysis (LDA), decision tree, support vector machine (SVM), wavelet transform (WT), Hilbert-Huang transform (HTT), deep learning mechanisms, and so on.

Some of the data-driven methods are based on deep neural networks for predicting the bearing RUL; they are so-called deep learning methods. Deep learning bearing RUL prediction methods are shown in [30] to have better RUL prediction accuracy than other data-driven methods. Therefore, only deep learning RUL prediction methods [30,31,32,33,34,35,36,37,38,39] are reviewed below. Their basic concept is to apply the deep neural network, which consists of an input layer, an output layer, and several hidden layers of neurons to analyze data for extracting data features and discovering knowledge hidden in data. The applied neural networks include the common deep neural network (DNN), convolutional neural network (CNN), recurrent neural network (RNN), deep autoencoder (DAE) network, LSTM (long short-term memory) network, and GRU (gated recurrent unit) network with or without certain feature transformation/extraction schemes. Every method will be detailed later.

This paper proposes two deep learning methods to predict the RUL of bearings. The methods have the advantageous end-to-end property that they take raw data as input and generate the predicted RUL directly. The first method is TSMC-CNN, which stands for the time series multiple channel convolutional neural network. The second method is TSMC-CNN-ALSTM, which stands for the TSMC-CNN integrated with the attention-based long short-term memory (ALSTM) network. The proposed methods divide a time series into multiple channels and take advantage of the CNN, the LSTM network, and the attention mechanism for boosting performance. The CNN performs well for extracting features from data; dividing a time series into multiple channels helps the CNN extract relationship among far-apart data points. The LSTM network is excellent for processing temporal data; the attention mechanism allows the LSTM network to focus on different features at different time steps for better prediction accuracy.

The open-accessed bearing operation data [40] collected by FEMTO-ST, a French research institute, on the PRONOSTIA platform [41] is used to evaluate the performance of the proposed methods. The evaluation results are also compared with those of related deep learning and data-driven methods, namely, the deep neural network (DNN), Gradient Boosting Decision Tree (GBDT), Support Vector Machine (SVM), BP neural network (BPNN), Gaussian regression (GR), and Bayesian Ridge (BR) methods, as described in [30]. The comparison results show that the proposed methods outperform the others in terms of the mean absolute error (MAE) and the root mean squared error (RMSE) of RUL prediction.

The rest of the paper is organized as follows. The PRONOSTIA datasets and several deep learning methods for predicting the bearing RUL are elaborated in Section 2. The proposed methods are described in Section 3. The performance evaluation and comparison results are shown in Section 4. At last, Section 5 gives some concluding remarks of this paper.

## 2. Related Work

**Figure 1:**
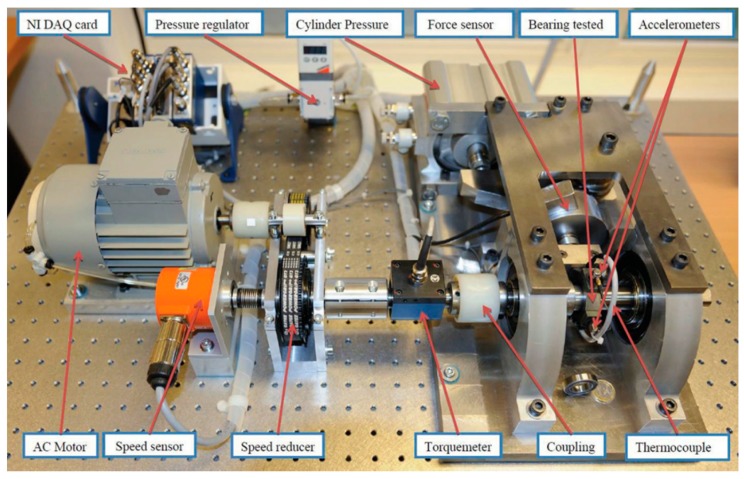
The PRONOSTIA platform [41].

**Figure 2:**
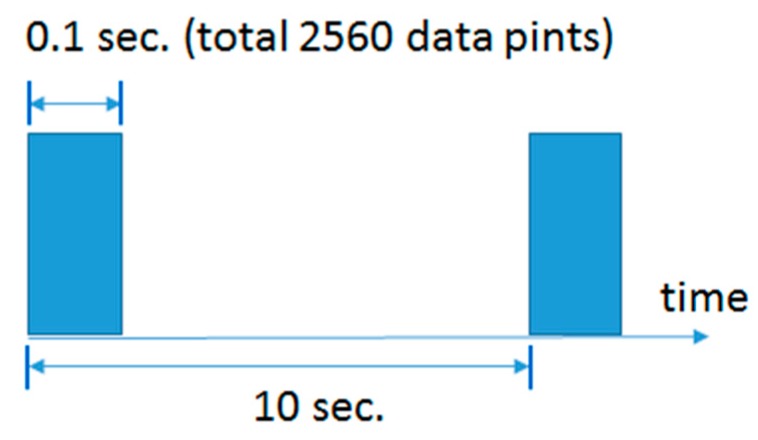
The data acquisition of the vibration sensor lasts for 0.1 s per 10 s.

**Figure 3:**
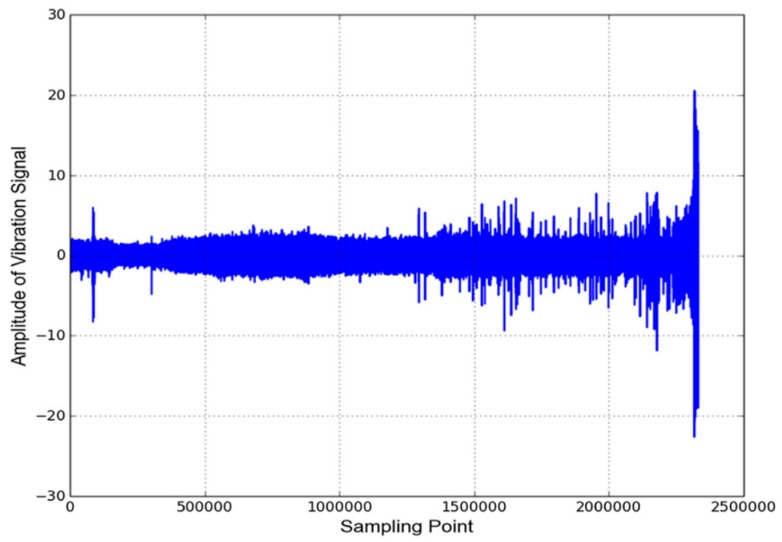
The PRONOSTIA time series data [41].

This section elaborates on deep learning methods [30,31,32,33,34,35,36,37,38,39] for predicting the bearing RUL, whose characteristics are shown in Table 1. PRONOSTIA datasets [40] are first described before the methods are detailed, as all the methods presented in this section apply PRONOTIA datasets for prediction accuracy evaluation. PRONOSTIA datasets are gathered on the PRONOSTIA platform, as shown in Figure 1. Most of the data come from two vibration sensors set alongside the x-axis and the y-axis of the bearing under accelerated degradation testing. A testing bearing is installed on the PRONOSTIA platform and rotates at the constant speed of 1800, 1650, or 1500 rpm. A constant payload weight of 4000 N, 4200 N, or 5000 N is applied to the testing bearing. When the amplitude of the testing bearing vibration signal exceeds 20 g, the bearing is regarded to be faulty. The dataset contains data of 17 testing bearings under three different testing conditions, as shown in Table 2. A vibration sensor acquires data with a frequency of 25.6 kHz, and the data acquisition lasts for 0.1 s every 10 s, as shown in Figure 2. Thus, there are 2560 data points for every 0.1 s of data acquisition. Figure 3 shows the collective vibration data or signals acquired.

Ren et al. [30] proposed a deep learning method that extracts time-domain and frequency-domain features of vibration signals and feeds them into a DNN for predicting the bearing RUL. The time-domain features extracted are: signal root mean square, crest factor, and kurtosis, and the frequency-domain feature extracted is the frequency spectrum partition summation, which is calculated on the basis of Fourier transformation of signals. The DNN has eight layers having 300, 200, 150, 100, 80, 50, 30, and 1 neuron(s), respectively. Uniform distribution is applied for weight parameter initialization. The activation function of middle layers (resp., the output layer) is the ReLU (resp., sigmoid) function. The loss function is the MAE and the optimizer to minimize the loss function is RMSprop. PRONOTIA datasets are applied for the prediction accuracy evaluation by partitioning the dataset as the training dataset and the test dataset. The percentage of the training dataset is 70%, 75%, 80%, 85%, 90%, or 95%. The prediction accuracy results are also compared with those of traditional data-driven methods, namely, the gradient boosting decision tree (GBDT), support vector machine (SVM), back propagation neural network (BPNN), Gaussian regression (GR), and Bayesian ridge (BR). The comparison results show that the DNN outperforms the other methods.

Guo et al. [31] proposed a deep learning method using the RNN-health indicator (RNN-HI) for estimating bearing RUL. The basic idea is to first combine six related-similarity (RS) features with eight time-frequency features to form an original feature set. One of the RS features is derived from eleven time-domain statistical features, including the mean, RMS, kurtosis, skewness, peak-to-peak, variance, entropy, crest factor, wave factor, impulse factor and margin factor. The other five RS features are derived according to the full frequency spectrum and four sub-bands frequency spectra, which are located in 0–12.8 kHz, 0–3.2 kHz, 3.2–6.4 kHz, 6.4–9.6 kHz and 9.6–12.8 kHz, respectively. And the eight time-frequency features are derived on the basis of the energy ratios of eight frequency sub-bands generated by performing Haar wavelet package transform with a three-level decomposition on vibration signals. The most sensitive features are then selected from the original feature set with monotonicity and correlation metrics. Finally, the selected features are fed into an RNN to construct the RNN-HI, which is actually the normalized RUL ranging between 0 and 1. It was shown in [31] that the method using RNN-HI has better prediction performance than the method using the classical self-organizing map-based health indicator (SOM-HI) [42].

Ren et al. [32] proposed a deep learning method using a CNN and a DNN to predict the RUL of bearings. First, the frequency spectrum of a time step is divided into *k* (say, *k* = 64) blocks, and the maximum spectrum amplitude of each block is derived to form a *k*-dimension eigenvector, named the spectrum-principal-energy-vector. Afterwards, eigenvectors of *k* time steps are combined together to form a *k* × *k* feature map to be fed into the CNN to generate an *l*-dimension (say, *l* = 360) feature. The *l*-dimension feature is then fed into the DNN for predicting the bearing RUL. Note that the CNN has three convolutional layers, three average pooling layers, and one flatten layer. The filters of the three convolutional layers are of the size 64 × 64, 14 × 14, and 6 × 6 with 40 channels using the ReLU activation function. The filters of the pooling layers are all of the size 2 × 2. The flatten (fully connected) layer has 360 neurons and uses the ReLU activation function. Also note that the DNN has an input layer of the size of 360 and six other layers with 200, 100, 50, 30, 8, and 1 neuron(s), respectively, with the ReLU activation function. The authors also proposed a smoothing mechanism to address the discontinuity problem occurring in the prediction results. The method proposed in [32] is shown to have better prediction accuracy than the method using the SVM and the method using the DNN with time-frequency wavelet transformation features.

Mao et al. [33] proposed a bearing RUL estimation method using the concept of the Hilbert–Huang transform (HHT) [43], CNN, and LSTM. First, the HHT is applied to vibration signals to obtain the HHT marginal spectrum as the input of the CNN. A new criterion, named support vector data (SVD) normalized correlation coefficient, is used to perform health state assessment and divide the whole bearing life into the normal state and the fast degradation state. More specifically, the sequence of data for the whole life of a bearing is divided into several sub-sequences. Then, the phase space reconstruction of each sub-sequence is performed by applying the Hankel matrix [44], whose singular value vector is used to assess the health state of the bearing. The two states are used as CNN output labels for training the CNN. The CNN has two convolutional layers and two max pooling layers whose filters are all of the size 2 × 2. The first and the second convolutional layers have 64 and 128 channels, respectively. The flatten layer has 25 neurons and uses the softmax function to output machine state classification. Note that the flatten layer is used as the deep feature representation of the vibration signals to be fed into the LSTM network for RUL prediction. The proposed method is compared with other methods, such as the SVM [45], linear regression (LR) [46], GPR [47], and extreme learning machine (ELM) [48], to show its superiority.

Ren et al. [34] proposed a deep learning method using a deep autoencoder (DAE) and a DNN for bearing RUL estimation. First, vibration signal features are extracted, including time domain, frequency domain, and time-frequency domain features. There are totally 36 time domain features for signals along the x axis and the y axis; they are the maximum, minimum, peak-peak, mean, root mean square root, skewed, standard deviation, absolute average, kurtosis, variance, coefficient of variation, crest factor, clearance factor, waveform factor, kurtosis coefficient, energy operator, pulse factor, and skew factor of signals. The 36 features are then fed into an 11-layer DAE for feature compression. The DAE has 36, 40, 30, 15, 9, 5, 9, 15, 30, 40, 36 neurons in each layer, respectively. The first 10 layers apply ReLU as the activation function, while the last layer, Sigmoid. Therefore, the 36 time domain features are now compressed into five features. By dividing the whole FFT (Fast Fourier Transformation) spectrum into six segments, 12 FSPS (frequency spectrum partition summation) frequency domain features are extracted for *x*-axis and *y*-axis vibration signals. Through the third-order wavelet decomposition, 16 time-frequency domain features are derived for *x*-axis and *y*-axis vibration signals. The five compressed time domain features, 12 frequency domain features, and 16 time-frequency domain features form the 33-tuple eigenvector to be fed into the DNN for RUL estimation. The DNN has nine layers with 33, 30, 25, 20, 15, 10, 6, 3, and 1 neuron(s) in each layer. The proposed method is compared with other methods, such as none-autoencoder DNN (NAE-DNN), DNN with FSPS (DNN/FSPS), and SVM to show its superiority in terms of the MSE of RUL prediction.

Hinchi et al. [35] proposed an end-to-end bearing RUL prediction method based on convolutional and LSTM neural networks. The term “end-to-end” means that the proposed method takes raw data as input and generate RUL prediction directly. That is to say, the method needs not to extract features from raw data manually; it needs not estimate a health indicator using a failure threshold for the RUL prediction, either. Specifically, the method predicts the RUL directly from raw vibration signals by stacking a convolutional layer, a global average pooling layer, and an LSTM layer. Note that the global average pooling layer also produces an auxiliary output, namely, the degradation percentage, by taking the dense layer. For regularizing the neural network, batch normalization is executed after each neural layer, and dropout is conducted for the input of the LSTM layer. The MAE of the RUL prediction is used as the lost function and the MAE of the predicted degradation percentage is used as an auxiliary loss function. The two loss functions are both minimized with the ADAM optimizer. The proposed method is compared with the method proposed by the Center for Advanced Life Cycle Engineering (CALCE) [14], which was the winner of the 2012 PHM data challenge competition [41] and is simply called the CALCE method later, to show that the proposed method has comparably good prediction accuracy.

Zhu et al. [36] proposed a bearing RUL prediction method based on the time frequency representation (TFR) and the multiscale convolutional neural network (MSCNN). The wavelet transformation is applied to vibration signals to derive their TFRs. As these TFRs have very high dimensions, a bilinear interpolation is applied to reduce their dimensions. The resized TFRs as well as their corresponding RUL labels are fed into the MSCNN for bearing RUL prediction. The MSCNN has three convolutional layers, two pooling layers, and one flatten layer. The structure of the MSCNN is similar to that of the normal CNN except that the latter puts features in the last convolutional layer into the flatten (fully connected) layer, while the latter puts features in the last convolutional layer as well as features in the pooling layer before the convolutional layer into the flatten layer, which is specifically called the multiscale (mixed) layer. The application of the MSCNN was shown to be very useful for bearing fault diagnosis by Ding et al. [49]. The proposed method is compared with three data-driven HI-based methods, namely, the RNN-HI method, the SOM-HI method and the SVR-HI method, to show its superiority in the RUL prediction accuracy.

Yoo et al. [37] proposed a deep learning method using the continuous wavelet transform and convolutional neural network-based health indicator (CWTCNN-HI) for predicting the bearing RUL. The Morlet-based continuous wavelet transform (CWT) is applied to vibration signals to extract the time—frequency features (i.e., the wavelet power spectrum). The features are then fed into a CNN to construct the CWTCNN-HI, whose value is between 0 and 1. The Gaussian process regression (GPR) then estimates the RUL on the basis of the HI. The *K*-means clustering algorithm is also used to cluster testing bearings by using the Dunn index to choose the best value of *K*, which is 5. Different clusters have different failure thresholds of the CWTCNN-HI for the RUL prediction. The proposed method is compared with four related methods proposed by Sutrisno et al. [14], Hong et al. [50], Guo et al. [31], and Lei et al. [51], respectively. It is better than the first three methods but is worse than the last method.

Li et al. [38] proposed a deep learning method for bearing RUL prediction. The kurtosis of vibration signals is used to determine the first predicting time (FPT), since it is generally agreed that the kurtosis is sensitive to incipient faults [13]. At a time step after the FPT, each of *N* sequences of vibration data associated with the time step is applied to the short-time Fourier transform (STFT) to obtain the time-frequency domain features. The features are then fed into a multi-scale CNN (MSCNN) for directly predicting the bearing RUL. The MSCNN has one max-pooling layer with the filter of size 1 × 2, and four convolutional layers (CLs), each with 5 filters of the size 3 × 3. The max-pooling layer is to reduce the dimension of the time-frequency domain features. The features in turn go through three CLs. The feature maps generated by the three layers are then concatenated to form multi-scale features. The multi-scale features go through the fourth CL to generate the high-level representation (HLR) of a sequence. The HLRs of the *N* sequences of data are then element-wise added and fed into an extra CL with one filter of the size 3 × 3 for the purpose of feature compression. The compressed features are finally flattened and fed into a fully connected layer with 128 neurons, followed by the output layer with one neuron to generate the predicted RUL. The CLs use the leaky ReLU as the activation function and adopts the dropout mechanism with the rate of 0.5 to avoid overfitting. The proposed method is compared with four alternative methods, namely, the DNN, SSL (Single Scale-Low), SSH (Single Scale-High), and NoFPT methods, in terms of prediction accuracy. The DNN has four fully connected layers with 128, 128, 64, and 1 neuron(s). For the SSL method, only one CL is applied to process each sequence of data. For the SSH method, only the features generated by the third CL is used as the HLR of a sequence. As for the NoFPT method, no FPT is determined and the degradation process is assumed to start at the beginning of machine operation. It is shown that the proposed method has better prediction performance than the others.

Ren et al. [39] proposed a deep learning method using the multi-scale dense gated recurrent unit (MDGRU) neural network for bearing RUL estimation. Similar to the study in [34], time domain, frequency domain, and time-frequency domain features are first derived from vibration signals. They are then fed into the restricted Boltzmann machine (RBM) network for the purpose of pre-training the MDGRU network. The MDGRU network consists of two feature layers, one multi-scale layer, several skip-GRU layers, and three dense layers. The feature layers are initialized by the RBM network, while other layers are initialized randomly. The features generated by the feature layer are fed into the multi-scale layers for choosing features of different time scales, which in turn are fed into the skip-GRU layers. Unlike traditional GRU layers, skip-GRU layers have a skip connection between two consecutive layers, which can maintain enough information while information passes through layers. The outputs of the skip-GRU layers are concatenated and fed into three dense layers for the purpose of the ensemble learning of the skip-GRU outputs of three different time scales. The last dense layer then outputs the predicted bearing RUL. The proposed method is compared with the SVM, random forest (RF), Bayesian regression (BR) methods. It is observed that the proposed method outperforms the others in the aspects of prediction accuracy.

## 3. Proposed Methods

This section presents the two proposed methods. They are end-to-end deep learning methods for directly inputting the raw vibration signals and outputting the RUL prediction. The methods are the TSMC-CNN method using the CNN with multiple-channel input, and the TSMC-CNN-ALSTM method based on the TSMC-CNN method and the attention-based LSTM network. The details of the methods are shown below.

### 3.1. TSMC-CNN

The TSMC-CNN method takes advantage of the CNN, which has very good performance in image classification. The CNN is powerful and efficient due to the characteristics of neural parameter sharing and sparsity of neural connections. A typical CNN consists of the input layer, several suites of convolutional layers and pooling layers, one or more fully connected layers, and finally the output layer. The convolutional layer uses filters (or kernels) sliding over the image to perform the convolution operation for extracting features, call feature maps. One filter generates one feature map, which corresponds to a channel to be fed into the following layer. Note that before the feature map is generated, an activation function, such as the ReLU function, is applied. The filter with the size of width *w* by length *l* slides over the image in the left-to-right and top-to-bottom manner with hyperparameters, such as the stride, which stands for the number of pixels to jump when the filter moves, and the padding, which stands for the number of zeros to be padded on the borders. The pooling layer also uses a filter to slide over an image map. It is used to subsample the image map (i.e., to shrink the size of the image map) without damaging the extracted features. Two common pooling layers are the max pooling layer, which returns the maximum value in the filter region, and the avg pooling layer, which returns the average of values in the filter region.

**Figure 4:**
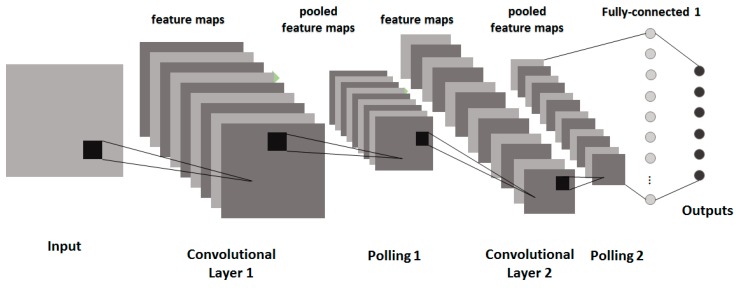
The network structure of a CNN.

After the convolutional and the pooling layers, are the fully connected layers (or dense layers or flatten layers). The image maps generated by the last pooling layer are first flattened. That is, they are transformed from the multiple-dimension shape into the one-dimension shape as a multi-tuple vector. To avoid overfitting, the dropout mechanism is applied in the dense layers. The multiple-tuple vector then goes through zero, one or more dense (i.e., fully connected) neuron layers, and finally the output layer. If the CNN is for the purpose of classification, then a softmax function is used to output the classification result. However, if the CNN is for the purpose of regression, then another activation function (e.g., the sigmoid function) is used to output the regression result. Figure 4 shows the illustrative diagram of a typical CNN [52] for the purpose of image classification.

**Figure 5:**
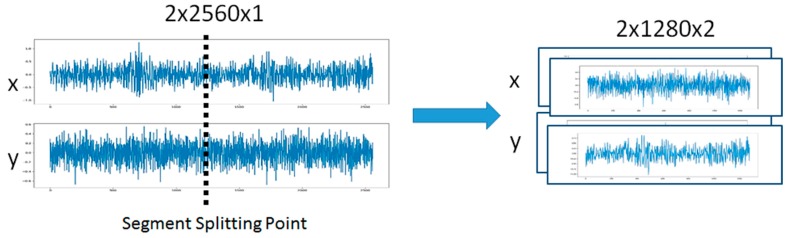
The time series of the 2 × 2560 × 1 shape (with 2 rows, 2560 columns, and 1 channel) is reshaped to be of the 2 × 1280 × 2 shape (with 2 rows, 1280 columns, and 2 channels).

**Figure 6:**
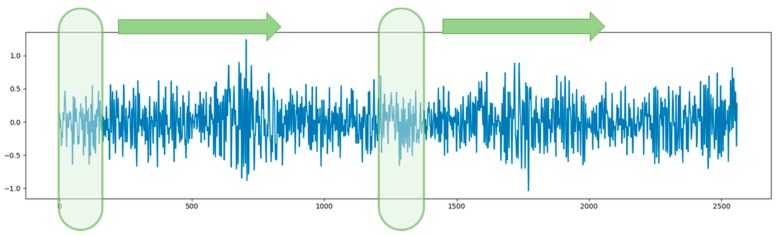
A two-channel filter slides at the first half part and the second half part at the same time.

The TSMC-CNN method takes an *N*-variate time series as input. The time series of length *L* is first divided into *K* segments, where *K* ≥ 2 and *L* is divisible by *K*. Note that if *L* is not divisible by *K*, the last data point can be repeated until the time series has a length that is divisible by *K*. The *K* segments of data are superimposed altogether as *K* channels of the input data of a CNN. This can help the CNN extract relationship among far-apart data points. For example, for *N* = 2, *L* = 2560, and *K* = 2, the time series is divided into two segments, each of which contains 1280 data points, each of which is in turn a 2-tuple (2-variate) vector, as shown in Figure 5. The 2-row, 2560-column, and 1-channel (2 × 2560 × 1) time series can be regarded as a 2-row, 1280-column, and 2-channel (2 × 1280 × 2) image to be input into a CNN. When the 2-channel time series data are fed into the CNN, a 2-channel filter slides at the first half part (i.e., the first channel) and the second half part (i.e., the second channel) of the time series at the same time to perform a convolution operation, as shown in Figure 6. Note that a convolution operation sums up corresponding results of all channels. This can help the convolution operation extract relationship among all channels. The results of convolution operations of multiple filters of a convolutional layer then form multiple-channel feature maps to go through a series of pooling layers and convolutional layers, if any, for extracting hierarchically higher-leveled relationship within a channel and/or among all channels. Therefore, the relationship among data points, which are either far or near, can be properly extracted.

### 3.2. TSMC-CNN-ALSTM

**Figure 7:**
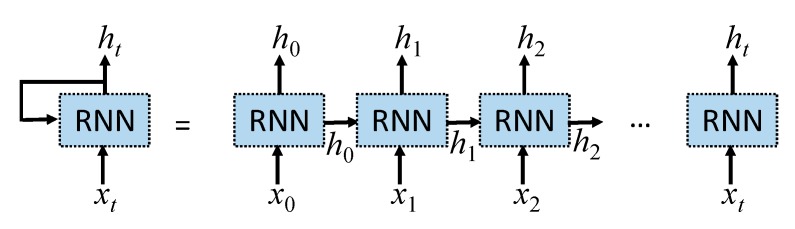
The structure of the RNN (**left**) and its application in processing time series data (**right**).

**Figure 8:**
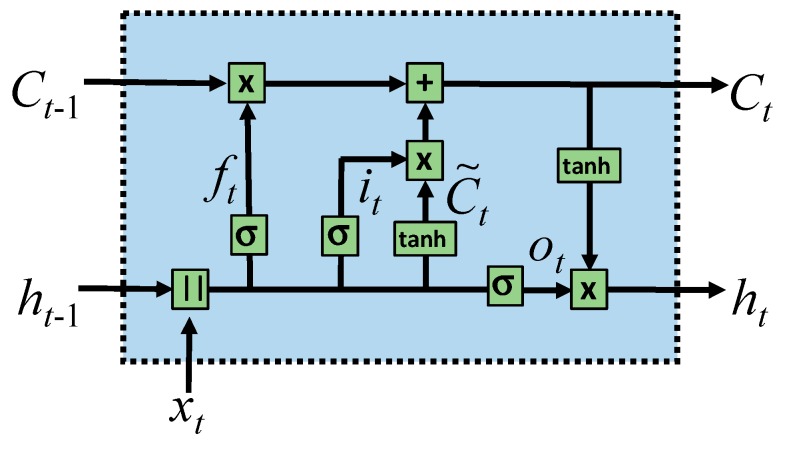
The structure of the LSTM network.

The TSMC-CNN-ALSTM method is based on both the TSMC-CNN method, as described previously, and the attention-based LSTM (ALSTM) network. The long short-term memory (LSTM) network [53] is a special type of the recurrent neural network (RNN) [54]. The RNN is suitable for processing time-series data, as it adds a loop to a neuron allowing the output at the current time step to be used as the input at the next time step. Figure 7 shows the structure of the RNN and its application in processing time series data, where *x_t_* and *h_t_* are the input data and the output at time step *t*, respectively. The RNN behaves as if it has “memory” to generate output according to data processed previously. It, nevertheless, has the problems of gradient vanishing and gradient exploding, making it difficult to reflect the dependency of input data far apart in the time series. The LSTM network, whose structure is shown in Figure 8, can mitigate the problems by including a memory cell and three gates: the input gate, the output gate, and the forget gate. Information can be added to or removed from the memory cell by the gates. Every gate has its own weights to be adjusted and thus the most important historical information can be stored in the memory cell for producing the most proper output. The equations for the LSTM network are Equations (1)–(6). In the equations, σ(·) stands for the sigmoid function, tanh(·) stands for the hyperbolic tangent function, and || stands for the concatenation operation. Moreover, Equations (1)–(6) are for the forget gate, input gate, memory cell, output gate, and output, respectively, where *W* stands for weights, and *b* stands for the bias.

(1) $$f_{t}=\sigma \left(W_{f}\cdot \left[h_{t-1}||x_{t}\right]+b_{f}\right)$$

(2) $$i_{t}=\sigma \left(W_{i}\cdot \left[h_{t-1}||x_{t}\right]+b_{i}\right)$$

(3) $${\tilde{C}}_{t}=\tan h\left(W_{C}\cdot \left[h_{t-1}||x_{t}\right]+b_{C}\right)$$

(4) $$C_{t}=f_{t}\cdot C_{t-1}+i_{t}\cdot {\tilde{C}}_{t}$$

(5) $$o_{t}=\sigma \left(W_{o}\cdot \left[h_{t-1}||x_{t}\right]+b_{o}\right)$$

(6) $$h_{t}=o_{t}\cdot \tan h\left(C_{t}\right)$$

**Figure 9:**
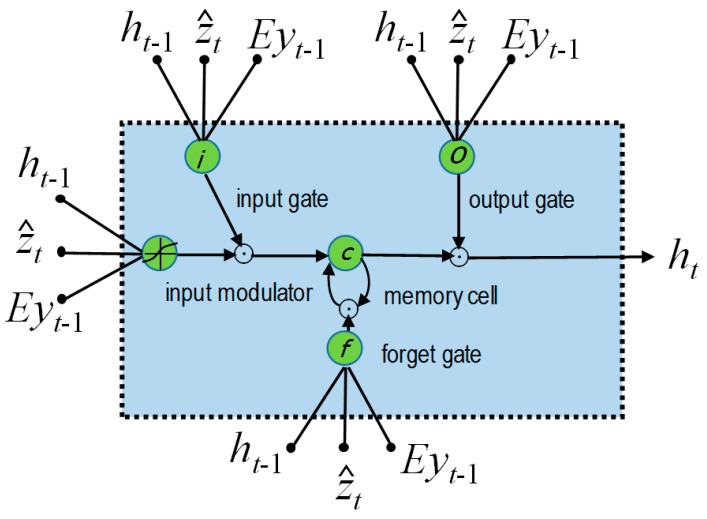
The structure of the ALSTM network.

The attention-based long short-term memory (ALSTM) network proposed in [55] can imitate the attention behavior of human vision. It and can focus on a certain area of the image with high concentration, while at the same time perceiving the surrounding image with low attention. It then can gradually adjust the focus over time. The ALSTM network adds a context vector for the purpose of inferring different attention degrees of different data features at different time points. The structure of the ALSTM, as shown in Figure 9, is similar to that of the LSTM. The equations for the ALSTM network are Equations (7)–(12). They are originally for the purpose of taking a raw image as input to generate a caption *y* encoded as a sequence of 1-of-*V* encoded words. That is, *y* = {*y*_1_, …, *y_C_*}, *y_i_* ∈ *R^V^*, where *V* is the size of the vocabulary and *C* is the length of the caption. A set *a* = {*a*_1_, …, *a_L_*} of *ld*-tuple vectors is first extracted from the image by an extractor (e.g., an CNN network), where *a_i_* ∈ *R^D^* is a *d*-tuple vector called the annotation vector. In Equation (7), $i_{t}$, $f_{t}$, $o_{t}$, and $g_{t}$ are for calculations of the input gate, forget gate, output gate, and potential information to be added into the memory cell, respectively. $T_{s,t}$: $R^{s}\to R^{t}$ is used to denote a simple affine transformation with parameters that are learned. *E* ∈ *R*^*m*×*V*^ is an embedding matrix, where *m* and *n* are the embedding and LSTM dimensionality, respectively. Moreover, $\hat{z_{t}}$ is the context vector at time point *t*, which is calculated by Equation (12) for capturing the visual information associated with a certain input location of the annotation vector. In Equations (8) and (9), ʘ stands for the element-wise multiplication. In Equation (10), $f_{att}$ is the attention function which is modeled by a multilayer perceptron to generate the weight $e_{ti}$ based on the annotation vector $a_{i}$ and the output $h_{t-1}$ of previous time step. Like the softmax function, Equation (11) transforms weight $e_{ti}$ into weight $\alpha_{ti}$ so that all weights have the total sum of 1. In Equation (12), *φ* is a function returning a single vector given the set of annotation vectors and their corresponding weights. There are different ways to realize the function *φ*. The details of the *φ* function are referred to Reference [55] for the sake of simplicity.

(7) $$\left(\begin{array}{c} \begin{array}{c} i_{t}\\ f_{t} \end{array}\\ \begin{array}{c} o_{t}\\ g_{t} \end{array} \end{array}\right)=\left(\begin{array}{c} \begin{array}{c} \sigma \\ \sigma \end{array}\\ \begin{array}{c} \sigma \\ tanh \end{array} \end{array}\right)T_{D+m+n,n}\left(\begin{array}{c} Ey_{t-1}\\ h_{t-1}\\ \hat{z_{t}} \end{array}\right)$$

(8) $$c_{t}=f_{t}ʘc_{t-1}+i_{t}ʘg_{t}$$

(9) $$h_{t}=o_{t}ʘ\tan h\left(c_{t}\right)$$

(10) $$e_{ti}=f_{att}\left(a_{i},h_{t-1}\right)$$

(11) $$\alpha_{ti}=\frac{\exp\left(e_{ti}\right)}{\sum_{k=1}^{L}\exp\left(e_{tk}\right)}$$

(12) $$\hat{z_{t}}=\emptyset \left(\left\{a_{i}\right\},\left\{\alpha_{i}\right\}\right)$$

**Figure 10:**
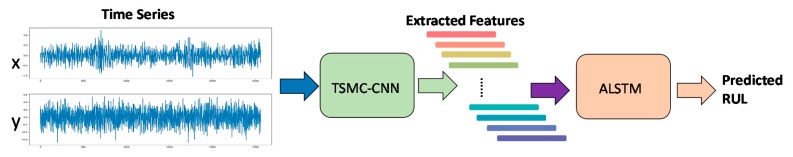
The network architecture of the TSMC-CNN-ALSTM method.

The TSMC-CNN-ALSTM method borrows the concept of the ALSTM network. It use the TSMC-CNN network to extract features from the time series, as shown in Figure 10. The extracted features are then fed into the ALSTM network so that features have different weights of focus at different time steps. Consequently, the prediction accuracy is further improved.

## 4. Performance Comparisons

The section shows results of performance evaluation and comparison. The PRONOSTIA bearing operation datasets [40] are used to evaluate the proposed methods’ performance. The evaluation results are also compared with those of related methods in terms of the MAE and the RMSE. The deep learning method using DNN [30] has been shown to be superior to five data-driven methods, namely, GBDT, SVM, BPNN, GR, and BR, as described in [30]. The proposed methods are compared with the DNN method [30] and the five data-driven methods. Other deep learning methods are not compared. This is because the deep learning methods described in this paper apply different experimental settings and not all of them are compared with one another. Some of them are even not compared with any deep learning methods; they are only compared with other traditional data-driven methods with experimental settings not clearly specified. The comparison results presented below show the proposed methods are superior to the DNN method, and certainly superior to the five data-driven methods compared.

As shown in Section 2, the PRONOSTA platform uses three operating conditions to accelerate degradation of bearings. The three operating conditions are 1800 rpm (rotating speed) and 4000 N (payload weight), 1650 rpm and 4200 N, and 1500 rpm and 5000 N. Two vibration sensors are set alongside the *x*-axis and the *y*-axis of the bearing. When the vibration signal amplitude exceeds 20 g, the experiment stops and RUL of the bearing is determined to be 0 at the corresponding time step. Four datasets using the conditions of 1650 rpm and 4200 N are used for performance evaluation. The four datasets correspond to the first four testing bearings with the maximum RUL of 7970, 23,110, 7510, and 2300 s, respectively.

The RUL of a bearing is assumed to decrease linearly from the maximum RUL value to 0. The RUL is first normalized to be between 0 and 1. Note that the normalized RUL matches well the sigmoid activation function of the last neuron layer adopted by the proposed methods, since the sigmoid function outputs values ranging from 0 to 1. The vibration sensor data are also normalized to be between 0 and 1. After normalization, every time series of length *L* (*L* = 2560) is divided into *K* channels, for *K* = 2, 3, 4, 5, and 8, to be input into the TSMC-CNN. The mean squared error (MSE) is used as the loss function and the adaptive moment estimation (Adam) algorithm is used as the optimizer to minimize the loss function for optimizing the TSMC-CNN.

**Figure 11:**
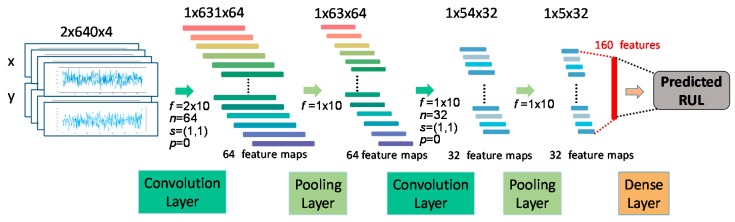
The TSMC-CNN with *K* channels (*K* = 4).

The structure of the TSMC-CNN with *K* = 4 is shown in Figure 11. The 2-row, 2560-column, and 1-channel (2 × 2560 × 1) time series is regarded as a 2-row, 640-column, and 4-channel (2 × 640 × 4) image to be input into the CNN. The first convolutional layer has *n* = 64 filters of the size 2 × 10 (kept by *f*) with stride = (1, 1) and no padding (*p* = 0). So, there are 64 feature maps of the size 1 × 631 (denoted as of the shape of 1 × 631 × 64). The activation function of the convolutional layer is the Leaky rectified linear unit (Leaky ReLU) with 0.2 being the leak or the ratio of negative values to be output. The first pooling layer is the average pooling layer with the filter of the size 1 × 10. So, the feature maps become of the shape 1 × 63 × 64. There then comes the second convolutional layer with 32 filters of the size 1 × 10 with stride = (1, 1) and no padding, and the second average pooling layer with the filter of the size of 1 × 10. Consequently, the final feature maps are of the shape of 1 × 5 × 32. The final feature maps are flattened as a 160-feature vector to be fed into the dense layer for predicting the RUL of bearings.

**Figure 12:**
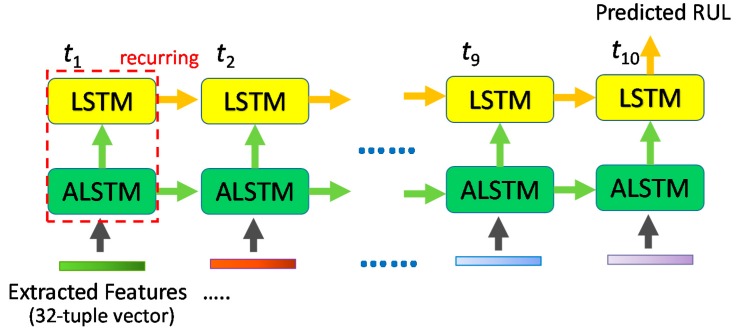
The ALSTM network adopted by the proposed TSMC-CNN-ALSTM method.

As shown in Figure 12, the TSMC-CNN-ALSTM method feeds the extracted features generated by the TSMC-CNN into the ALSTM network. Note that an LSTM layer is put on top of the ALSTM layer (or say an LSTM layer follows the ALSTM layer) to improve the prediction accuracy. For the case of the TSMC-CNN-ALSTM method, there are little adjustment of the TSMC-CNN convolutional layers and polling layers. To be more precise, the filter of the first convolutional layer is changed to of the size 2 × 30 (still 64 filters of four channels, stride = (1, 1) and no padding) to generate 64 feature maps of the shape 1 × 611 × 64. The first pooling layer is unchanged (with the size 1 × 10) and is to generate 64 feature maps of the shape 1 × 61 × 64. The second convolutional layer is unchanged and still has 32 filters of the size 1 × 10 to generate 32 feature maps of the shape 1 × 52 × 32. The second pooling layer is changed to have the filter of the size 1 × 5 to generate 32 feature maps of the shape 1 × 10 × 32, which is the extracted features to be fed into the ALSTM network. The extracted features are regarded as a time series of 10 data points, each of which is a 32-tuple vector. The ALSTM network, along with the on-top LSTM network, generates RUL prediction for every 10 data points.

Like the study of [30], this paper also adopts 6 ratios of training data and test data. They are 70–30%, 75–25%, 80–20%, 85–15%, 90–10%, and 90–5% of training data and test data. Performance evaluations are conducted by randomly splitting data 20 times for every ratio of training data and test data. Afterwards, average MAE and RMSE are derived to be compared with those of related methods.

The comparison results of the proposed methods with related methods are shown in Table 3. We can see that the TSMC-CNN method outperforms all other existing methods in terms of the MAE and the RMSE for 2, 3, 4, 5, and 8 channels of input data. This is because dividing data into multiple channels makes the CNN capable of extracting relationship between data points that are either near or far apart in time series. We can also observe that the TSMC-CNN method has the best performance when taking input data of four channels. Therefore, the TSMC-CNN with four channels is adopted by the TSMC-CNN-ALSTM method to further improve performance. We can observe that the TSMC-CNN-ALSTM method is even better than the TSMC-CNN method. However, the TSMC-CNN-ALSTM method takes more time in training.

**Figure 13:**
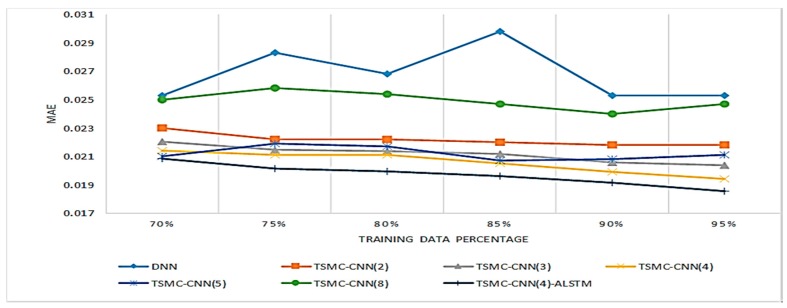
Comparisons of DNN, TSMC-CNN and TSMC-CNN-ALSTM with different numbers of channels in terms of the MAE.

**Figure 14:**
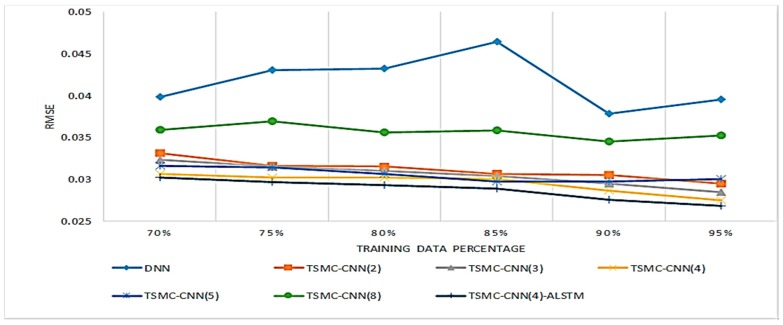
Comparisons of DNN, TSMC-CNN and TSMC-CNN-ALSTM with different numbers of channels in terms of the RMSE.

Figure 13 and Figure 14 further show the comparison results of the proposed methods with the DNN method proposed in [30]. The comparison assumes the TSMC-CNN method adopts 2, 3, 4, 5, and 8 channels, and the TSMC-CNN-ALSTM method adopts four channels. The DNN method adopts the neural network architecture consisting of 8 hidden layers having 300, 200, 150, 100, 80, 50, 30, and 1 neurons, respectively. The middle layers use rectified linear unit (ReLU) function, while the last layer uses the sigmoid function as the activation function. The MSE is taken as the loss function and the RMSProp algorithm is selected as the optimizer. It is obvious that the proposed methods are significantly better than the DNN method in terms of the MAE and the RMSE.

## 5. Conclusions

This paper proposes two deep learning methods, called TSMC-CNN and TSMC-CNN-ALSTM, for RUL prediction of bearings. The methods have the advantageous end-to-end property that they take raw data as input and generate the predicted RUL directly. The time series data of bearings are first divided into multiple channels to be fed into the CNN for predicting the RUL of bearings directly. The CNN performs well in extracting a relationship between data points; dividing a time series into multiple channels helps the CNN extract relationship among far-apart data points. The TSMC-CNN method thus has good prediction performance. The LSTM network is excellent for processing temporal data, and the attention mechanism allows the LSTM network to focus on different features at different time steps. Therefore, the TSMC-CNN-ALSTM method, which integrates the TSMC-CNN with the ALSTM network, is even better than the TSMC-CNN method. The PRONOSTIA bearing operation datasets [40] are used for performance evaluation. The proposed methods are also compared with the DNN, GBDT, SVM, BPNN, GR, and BR methods, as described in [30]. The comparison results show that the proposed methods are better than others in terms of the MAE and the RMSE of the RUL prediction.

The investigations of the bearing RUL prediction problem in this paper show again that the proposed deep learning methods outperform other kinds of data-driven methods. In the future, we plan to do a comprehensive performance comparison study of the proposed methods with more deep learning RUL prediction methods. Furthermore, we also plan to apply different deep learning methods, their integration, and/or their variants to solve the problem for achieving better prediction performance.

## Figures and Tables

**Table 1:** Characteristics of deep learning methods for RUL prediction of bearings.

Ref.	Networks	Features	Methods Compared	Year
Ren et al. [30]	DNN	TDF + FDF	GBDT, SVM, BPNN, GR, BR	2017
Guo et al. [31]	RNN (LSTM)	RNN-HI (RS + TFDF)	SOM-HI	2017
Ren et al. [32]	CNN + DNN	Spectrum-Principal-Energy-Vector	SVM and DNN with wavelet features	2018
Mao et al. [33]	CNN + LSTM	Hilbert-Huang Transform Marginal Spectrum	SVM, LR, GPR, ELM	2018
Ren et al. [34]	DAE + DNN	TDF + FDF + TFDF	NAE-DNN, DNN/FSPS, SVM	2018
Hinchi et al. [35]	Convolutional LSTM	Raw data	CALCE	2018
Zhu et al. [36]	MSCNN	Time-Frequency-Representation (TFR)	RNN-HI, SOM-HI, SVR-HI	2018
Yoo et al. [37]	CNN	Continuous Wavelet Transform and CNN-based Health Indicator (CWTCNN-HI)	CALCE and methods proposed by Hong et al. Guo et al. and Lei et al.	2018
Li et al. [38]	MSCNN	Multi-scale High-level Representation	DNN, SSL, SSH, NoFPT	2019
Ren et al. [39]	RBM + MDGRU	TDF + FDF + TFDF	SVM, RF, BR	2019

TDF: Time Domain Feature; FDF: Frequency Domain Feature; TFDF: Time-Frequency Domain Feature.

**Table 2:** PRONOSTIA dataset details.

Conditions	Load (N)	Speed (rpm)	Bearings
1	4000	1800	Bearing1-1, Bearing1-2, Bearing1-3, Bearing1-4, Bearing1-5, Bearing1-6, Bearing1-7
2	4200	1650	Bearing2-1, Bearing2-2, Bearing2-3, Bearing2-4, Bearing2-5, Bearing2-6, Bearing2-7
3	5000	1500	Bearing3-1, Bearing3-2, Bearing3-3

**Table 3:** Comparisons of the proposed methods with related methods.

	Training Data (%)	70%	75%	80%	85%	90%	95%
Methods							
GBDT							
MAE:		0.0378	0.0393	0.0378	0.0303	0.0378	0.0378
RMSE:		0.056	0.0547	0.0564	0.0552	0.0569	0.0866
SVM							
MAE:		0.06	0.058	0.057	0.057	0.058	0.059
RMSE:		0.081	0.0797	0.0785	0.0787	0.079	0.0866
BPNN							
MAE:		0.1075	0.1106	0.1121	0.1121	0.1196	0.1181
RMSE:		0.1428	0.1471	0.1457	0.1485	0.1514	0.1557
GR							
MAE:		0.059	0.0575	0.0575	0.0575	0.0575	0.059
RMSE:		0.0927	0.0915	0.0947	0.092	0.0922	0.0925
BR							
MAE:		0.1368	0.1396	0.1393	0.1391	0.1403	0.1385
RMSE:		0.17	0.1728	0.1714	0.1714	0.1728	0.1714
DNN							
MAE:		0.0253	0.0283	0.0268	0.0298	0.0253	0.0253
RMSE:		0.0398	0.043	0.0432	0.0464	0.0378	0.0395
TSMC-CNN (2)							
MAE:		0.023	0.0222	0.0222	0.022	0.0218	0.0218
RMSE:		0.0331	0.0316	0.0315	0.0306	0.0305	0.0295
TSMC-CNN (3)							
MAE:		0.022	0.0214	0.0214	0.0212	0.0206	0.0204
RMSE:		0.0323	0.0315	0.031	0.0304	0.0295	0.0285
TSMC-CNN (4)							
MAE:		0.0214	0.0211	0.0211	0.0205	0.0199	0.0194
RMSE:		0.0306	0.0302	0.0302	0.03	0.0286	0.0275
TSMC-CNN (5)							
MAE:		0.021	0.0219	0.0217	0.0207	0.0208	0.0211
RMSE:		0.0316	0.0314	0.0306	0.0297	0.0297	0.03
TSMC-CNN (8)							
MAE:		0.025	0.0258	0.0254	0.0247	0.024	0.0247
RMSE:		0.0359	0.0369	0.0356	0.0358	0.0345	0.0352
TSMC-CNN (4)-ALSTM							
MAE:		0.0208	0.0201	0.0199	0.0196	0.0192	0.0186
RMSE:		0.0302	0.0296	0.0293	0.0289	0.0275	0.0268

## Nomenclature

Adam	adaptive moment estimation
AI	artificial intelligence
ALSTM	attention-based long short-term memory
BPNN	backpropagation neural network
BR	Bayesian regression
CALCE	center for advanced life cycle engineering
CL	convolutional layer
CNN	convolutional neural network
CWT	continuous wavelet transform
CWTCNN-HI	continuous wavelet transform and CNN-based health indicator
DAE	deep autoencoder
DNN	deep neural network
ELM	extreme learning machine
FDF	frequency domain feature
FFT	fast Fourier transformation
FPT	first predicting time
FSPS	frequency spectrum partition summation
GBDT	gradient boosting decision tree
GPR	Gaussian process regression
GR	Gaussian regression
GRU	gated recurrent unit
HHT	Hilbert-Huang transform
HLR	high-level representation
Leaky ReLU	leaky rectified linear unit
LDA	linear discriminant analysis
LR	linear regression
LS-SVR	least squares-support vector regression
LSTM	long short-term memory
MAE	mean absolute error
MDGRU	multi-dimensional gated recurrent units
MSCNN	multi-scale CNN
MSE	mean squared error
PCA	principal component analysis
PHM	prognostics and health management
RBM	restricted Boltzmann machine
ReLU	rectified linear unit
RF	random forest
RMS	root mean square
RMSE	root mean squared error
RNN	recurrent neural network
RNN-HI	RNN-health indicator
RS	related-similarity
RUL	remaining useful life
SOM-HI	self-organizing map based health indicator
SSH	single scale-high
SSL	single scale-low
STFT	short-time Fourier transform
SVD	support vector data
SVM	support vector machine
SVR-HI	support vector regression health indicator
TDF	time domain feature
TFDF	time-frequency domain feature
TFR	time frequency representation
TSMC	time series multiple channel
WT	wavelet transform
